# Atomic Chromium Coordinated Graphitic Carbon Nitride for Bioinspired Antibiofouling in Seawater

**DOI:** 10.1002/advs.202105346

**Published:** 2022-01-20

**Authors:** Qiang Luo, Yilan Li, Xiaobing Huo, Linqian Li, Yinqiao Song, Shipeng Chen, Hong Lin, Ning Wang

**Affiliations:** ^1^ State Key Laboratory of Marine Resource Utilization in South China Sea Hainan University Haikou 570228 P. R. China; ^2^ State Key Laboratory of New Ceramics & Fine Processing School of Material Science and Engineering Tsinghua University Beijing 100084 P. R. China

**Keywords:** biofouling, carbon nitride, hydrogen peroxide, hypobromous acid, single‐atom

## Abstract

Artificial nanozymes exerting enzyme functionality are recognized as promising alternatives of natural enzymes in biomimetic chemistry. Natural haloperoxidases that utilize hydrogen peroxide (H_2_O_2_) to catalytically convert halide into strong biocidal hypohalous acid hold great promise for thwarting biofouling, while their practical application remains highly questionable as instability of natural enzymes and inadequate H_2_O_2_. Herein a semiconducting nanozyme consisting of chromium single atoms coordinated on carbon nitride (Cr‐SA‐CN) that performs bifunctional roles of nonsacrificial H_2_O_2_ photosynthesis and haloperoxidase‐mimicking activity for antibiofouling is constructed. Such nanozyme is capable of generating H_2_O_2_ from water and O_2_ upon visible‐light illumination, and then sustainably self‐supplying H_2_O_2_ for haloperoxidase‐mimicking reaction in a sequential manner. This dual‐activity Cr‐SA‐CN overcomes H_2_O_2_ dilemma and yields hypobromous acid continuously, inducing remarkable bactericidal capability. When used as an eco‐friendly coating additive, it is successfully demonstrated that Cr‐SA‐CN enables an inert surface against marine biofouling. Thereby, this study not only illustrates an attractive strategy for antibiofouling but also opens an avenue to construct valuable nanoplatform with multifunctionality for future applications.

## Graphical abstract

A system, namely chromium single atoms coordinated on carbon nitride, is designed. Chromium incorporation endows the system with coupled bifunctions of hydrogen peroxide photosynthesis and haloperoxidase‐mimicking activity, enabling remarkable bactericidal capability and impressive antibiofouling performance. This multifunctional biomimetic design surmounts the natural haloperoxidases, paving a way for constructing advanced nanomaterials for a variety of antibiofilm processes.

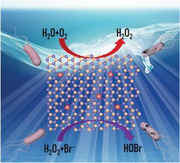

## 1 Introduction

Natural enzymes are powerful functional biocatalysts for many important biological processes with exceptional substrate specificity and catalytic efficiency.^[^ ^1^, ^2^, ^3^, ^4^, ^5^ ^]^ Unfortunately, natural protein enzymes suffer from high cost, intrinsic instability, and difficult storage. To circumvent these drawbacks, artificial nanozymes, with intrinsic enzyme‐like characteristics, have been devoted to mimicking natural enzymes in biomimetic chemistry.^[^ ^6^, ^7^, ^8^, ^9^, ^10^ ^]^ In nature, biofilm is an ubiquitous problem for maritime and aquatic industries with severely economic and ecological penalties.^[^ ^11^, ^12^, ^13^, ^14^ ^]^ Conventional antibiofouling strategies based on releasing persistently toxic metal biocides into water/seawater cause unacceptable environmental impacts.^[^ ^15^, ^16^, ^17^ ^]^ Marine algae, such as *Delisea pulchra*, have evolved fascinating defense strategies against biofilm colonization by secreting vanadium haloperoxidases.^[^ ^11^, ^18^, ^19^ ^]^ Such natural enzyme can generate strong biocidal but environmentally friendly hypohalous acid (HOBr, HOCl) through catalytic oxidation of halide with environmental hydrogen peroxide (H_2_O_2_).^[^ ^20^, ^21^, ^22^ ^]^ Artificial vanadium and cerium oxide nanozymes have been developed for combating biofilm by mimicking naturally occurring defense mechanisms of vanadium haloperoxidases, showing superior advantage compared to conventional highly toxic heavy metal biocides.^[^ ^23^, ^24^ ^]^ Nevertheless, the low level of environmental H_2_O_2_ (a typical concentration of ≈100 × 10^−9^ m in seawater)^[^ ^25^ ^]^ is the major bottleneck for haloperoxidase‐mimicking nanozyme to generate hypohalous acids sufficiently. To this end, it is imperative to create a H_2_O_2_ generating system that provides a sustainable and sufficient H_2_O_2_ supply for haloperoxidase‐like nanozyme.

The traditional routes for H_2_O_2_ generation, such as anthraquinone oxidation and noble metal‐based electrochemical synthesis,^[^ ^26^, ^27^ ^]^ need high energy input and harsh reaction conditions. Alternatively, H_2_O_2_ can be produced from photocatalytic two‐electron O_2_ reduction in combination with four‐electron water oxidation, providing a straightforward way for green H_2_O_2_ production with ambient reaction environment.^[^ ^28^, ^29^ ^]^ The major challenges for H_2_O_2_ photosynthesis are the low catalytic activity, poor reaction selectivity, and essential charge sacrificial agent. As for haloperoxidase‐mimicking nanozyme, if the production and utilization of H_2_O_2_ is not in the same compartment, meaning that an additional transport barrier need to be overcome. Cascade nanozymes that confine sequential or concurrent reactions in a single compartment can minimize mass‐transfer barriers and thus enhance local concentration of intermediates.^[^ ^30^ ^]^ However, designing efficient cascade nanozymes with synergic and complementary function in a single scaffold is very challenging, and requires the operating reaction conditions to be mutually compatible.^[^ ^31^ ^]^ Until now, no examples of such system involving in H_2_O_2_ production and haloperoxidase mimics has been explored.

Graphitic carbon nitride (CN) is a promising photocatalytic material due to its biocompatibility, photostability, and tailorability.^[^ ^32^ ^]^ Nevertheless, CN has a very poor photoactivity for H_2_O_2_ production in the absence of essential sacrificial agent.^[^ ^33^ ^]^ Chemical functionalization could endow CN with potentially a wide range of tunability in selectivity and activity.^[^ ^34^ ^]^ We thus reasoned that the installation of suitable active sites into CN could be a distinguished strategy for creating nanozymes with bifunctional roles of photocatalytic H_2_O_2_ generation and haloperoxidase‐mimicking ability. Encouragingly, single‐atom materials featuring maximized atomic utilization efficiency and unique coordination environment exhibit tremendous potential in heterogeneous catalysis for boosting desired catalytic pathways.^[^ ^35^, ^36^ ^]^

**Scheme 1:**
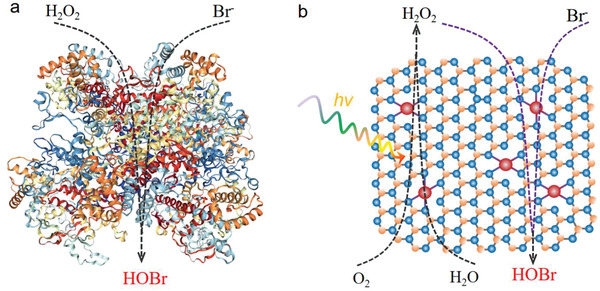
a) Bromide oxidation with H_2_O_2_ on a natural vanadium bromoperoxidase (RCSB PDB, 1QHB). b) Schematic illustration of the cascade reactions on an artificial bifunctional Cr‐SA‐CN nanozyme: photocatalytic H_2_O_2_ production from water and O_2_, and in situ self‐supply of H_2_O_2_ for bromide oxidation reaction.

In principle, the first step for H_2_O_2_ production in single atomic photocatalysts is the O_2_ activation by charge transfer from the metal center to O_2_, resulting in the formation of metal‐superoxo complexes,^[^ ^37^ ^]^ in which the combination of metal ions and ligands regulates the redox reactivity of the photocatalyst. In the first‐row of the *d*‐block metals, the Cr(III)‐superoxo complex experiences both hydrogen and oxygen atom transfer reactions.^[^ ^38^ ^]^ It is therefore highly desirable to develop Cr‐based nanozymes for O_2_ or H_2_O_2_‐mediated reactions. In this study, we report the first semiconducting single atom nanozyme by anchoring Cr single atoms on CN (Cr‐SA‐CN) with bifunctional roles of H_2_O_2_ photosynthesis and haloperoxidase‐mimicking capacity. Under visible light illumination, Cr‐SA‐CN with atomically dispersed Cr single atoms induces photocatalytic H_2_O_2_ production from water/seawater and O_2_, and the photogenerated H_2_O_2_ subsequently serves for the two‐electron Br^−^ oxidation to generate HOBr on the same Cr‐SA‐CN in an uninterrupted manner (**Scheme** **1**). Such cascade catalytic process exhibits outstanding broad‐spectrum antibacterial capability. Furthermore, field tests verify that the bifunctional Cr‐SA‐CN demonstrates impressive ability against biofouling in seawater.

## 2 Results and Discussion

### 2.1 Design and Characterization of Cr‐SA‐CN

**Figure 1:**
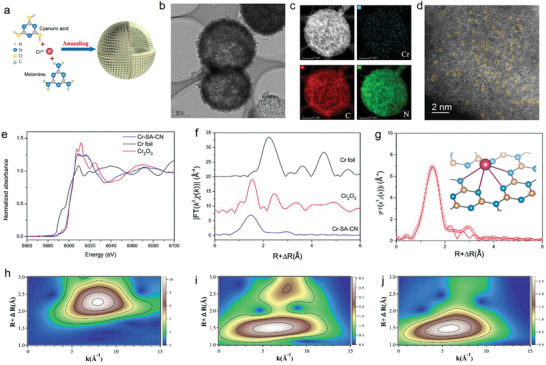
a) Schematic illustration of the synthesis of Cr‐SA‐CN. b) TEM image, c) EDS mapping, and d) aberration‐corrected HAADF‐STEM image of Cr‐SA‐CN. e) The normalized Cr K‐edge XANES spectra and f) *k* ^3^‐weighted Cr K‐edge Fourier transform EXAFS spectra of Cr‐SA‐CN and reference samples of Cr foil and Cr_2_O_3_. g) Experimental and fitting Cr K‐edge EXAFS curves of Cr‐SA‐CN, inset of g) is the schematic Cr‐N_4_ structure of Cr‐SA‐CN. The WT‐EXAFS plot of h) Cr foil, i) Cr_2_O_3_, j) Cr‐SA‐CN.

A thermal polymerization strategy was employed for the preparation of Cr‐SA‐CN (**Figure** **1**a). Typically, chromium ions from chromium nitrate were first precoordinated with melamine to form chromium‐melamine complex. Cyanuric acid was subsequently introduced into chromium‐melamine solution to obtain the white precipitate. The resulting solid was thermally polymerized under N_2_ atmosphere to form Cr‐SA‐CN. The coordination‐unsaturated N atoms in CN could offer sufficient coordination sites for isolating Cr atoms during thermal polymerization.^[^ ^39^ ^]^ X‐ray diffraction pattern (XRD) did not show any Cr‐containing crystal phases but only distinct peaks at 13.3° and 27.2° arising from CN (Figure S1, Supporting Information). The absorption band in the region of ≈1200–1650 and 3000–3500 cm^−1^, as well as the peak at 810 cm^−1^ in the Fourier transform infrared spectroscopy (FTIR) spectrum of CN represented the typical C—N, N—H, and triazine stretching vibration, respectively, confirming the typical structure of graphitic CN (Figure S2, Supporting Information).^[^ ^40^ ^]^ The FTIR spectrum of Cr‐SA‐CN exhibited the similar characteristic vibration peaks with CN, revealing that the overall structure of CN was maintained after Cr incorporation. Transmission electron microscopy (TEM) (Figure 1b) and scanning electron microscopy (SEM) (Figure S3, Supporting Information) indicated that Cr‐SA‐CN exhibited a hollow spherical structure with an average size distribution of about 3 µm. The Brunauer–Emmett–Teller (BET) surface area was characterized to be 75 m^2^ g^−1^ (Figure S4, Supporting Information). The energy‐dispersive X‐ray spectroscopy (EDS) demonstrated that Cr was homogeneously distributed throughout the CN framework (Figure 1c). The aberration‐corrected high‐angle annular dark‐field scanning transmission electron microscopy (HADDF‐STEM) in Figure 1d revealed the atomic Cr distribution. The full X‐ray photoelectron spectroscopy (XPS) spectrum demonstrates the appearance of Cr element in Cr‐SA‐CN (Figure S5, Supporting Information). The peaks at 577.8 and 588.1 eV could be attributed to 2p_1/2_ and 2p_3/2_ peaks of Cr^3+^.^[^ ^41^ ^]^ The high‐resolution N 1s spectrum was deconvoluted into pyridinic N (398.7 eV), Cr‐N (399.7 eV), and pyrrolic N (400.3 eV), respectively, suggesting that Cr atoms can coordinate with N to form Cr–N*_x_* moieties. The mass loading of Cr was ≈0.95 wt% as determined by inductively coupled plasma atomic emission spectrometry (ICP‐AES).

X‐ray absorption near‐edge spectroscopy (XANES) and extended X‐ray fine structure (EXAFS) were used to explore the electronic structure and coordination environment of single‐atomic Cr species. The normalized Cr *K*‐edge absorption of Cr‐SA‐CN exhibited higher energy than Cr foil but similar edge shape with Cr_2_O_3_ reference (Figure 1e), indicating that Cr atoms had an average oxidation state of ≈3. However, XANES curve of Cr‐SA‐CN obviously deviated from that of reference Cr_2_O_3_, indicating that the free electrons were partially depleted by coordinated N atoms. The Fourier transform EXAFS spectrum in *R* space for Cr‐SA‐CN was also obviously different from that for Cr foil and Cr_2_O_3_ references, with only one prominent peak located at 1.5 Å arising from the first shell Cr–N coordination (Figure 1f). No peak assigned to Cr–Cr coordination at longer distances (2.2 Å) can be observed, giving solid evidence for the formation of atomically dispersed Cr and a low degree of disorder for the coordination structure around Cr. The related fitting curve was displayed in Figure 1g and the fitting structural parameters was listed in Table S1 (Supporting Information). The result indicated that the first shell coordination number of Cr atoms was 4.2, confirming that one Cr atom was coordinated by fourfold N atoms, forming a Cr‐N_4_ moiety with an average bond length of 2 Å. The wavelet transform (WT)‐EXAFS of the Cr‐SA‐CN was then carried out. It was observed that Cr‐SA‐CN exhibited strong WT maximum focused at 5.8 Å^−1^ (Figure 1j), which was sharply distinguished from WT contour plots of Cr foil (Cr–Cr, *k* = 8.0 Å^−1^) and Cr_2_O_3_ (Cr–O, *k* = 6.2 Å^−1^) (Figure 1h,i). This result further confirmed the atomic dispersion of Cr on CN framework.

### 2.2 Photocatalytic H_2_O_2_ Generation

**Figure 2:**
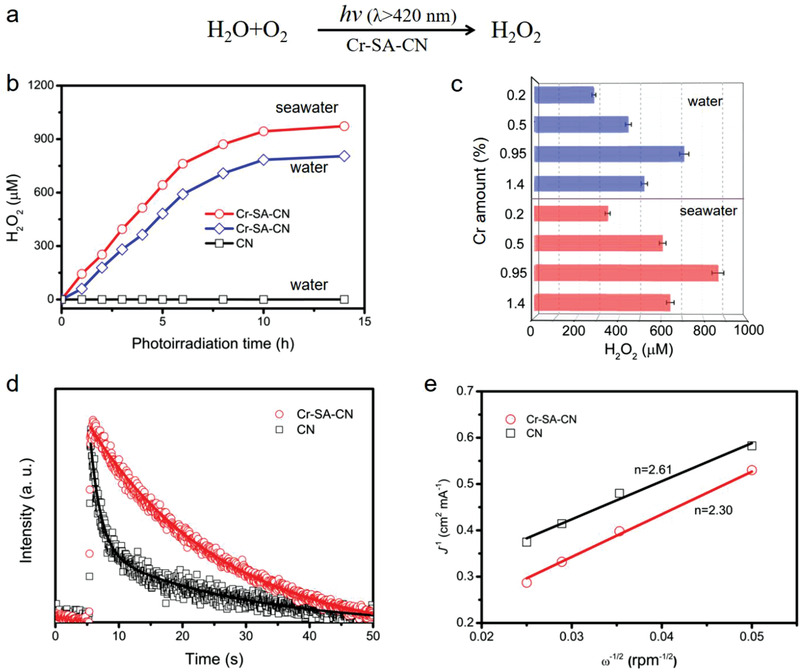
a) Photocatalytic H_2_O_2_ production from H_2_O and O_2_ under visible‐light illumination. b) Time course of H_2_O_2_ photosynthesis on Cr‐SA‐CN and CN in pure water and real seawater under AM 1.5G illumination (*λ* > 420 nm). c) H_2_O_2_ production on Cr‐SA‐CN with different Cr loading after 8 h of photoirradiation. d) Time‐resolved photoluminescence decay of Cr‐SA‐CN and CN samples with an excitation wavelength of 375 nm. e) Koutecky–Levuch plots of Cr‐SA‐CN and CN at –0.8 V versus Ag/AgCl, the current density *J* was extracted from the linear sweep voltammetry curves measured on RDE at different rotating speeds.

The photocatalytic activity of Cr‐SA‐CN was first evaluated in O_2_‐saturated pure water without additional scavenger (**Figure** **2**a). The time‐dependent change in the formed amount of H_2_O_2_ under AM 1.5G simulated sunlight irradiation (*λ* > 420 nm) was displayed in Figure 2b. Pristine CN generated negligible H_2_O_2_ amount (less than 1 × 10^−6^ m), suggesting its very poor photocatalytic activity.^[^ ^33^ ^]^ By comparison, H_2_O_2_ can be rapidly produced over Cr‐SA‐CN under identical experimental conditions, and the production rate of H_2_O_2_ was almost linear with time at the early stage of light irradiation. The amount of formed H_2_O_2_ after 8 h light illumination was about 870 times higher than that obtained with pristine CN, highlighting the unique advantage of atomically dispersed Cr active sites. More importantly, Cr‐SA‐CN also showed excellent photocatalytic activity in O_2_‐saturated seawater, and its photocatalytic H_2_O_2_ generation in seawater was about 1.3–1.5 times than that in pure water, suggesting that the large number of interfering ionics in seawater promoted the efficient utilization of photogenerated carriers.^[^ ^42^ ^]^ It is worth emphasizing that this is the first report on H_2_O_2_ photosynthesis from seawater over single‐atom materials. When comparing Cr‐SA‐CN photocatalysts with different Cr amounts, all Cr‐SA‐CN samples exhibited significant H_2_O_2_ production (Figure 2c). Of the catalysts Cr‐SA‐CN with 0.95 wt% Cr loading produced the highest amount of H_2_O_2_, while the sample containing a larger amount of Cr showed decreased activity despite its narrower bandgap (Figure S6, Supporting Information). The inferior catalytic activity of Cr‐SA‐CN with 1.4 wt% Cr loading can be ascribed to the unfavorable valence band edge (1.21 eV) for water oxidation, as determined by Mott–Schottky plots. In addition, the formation of 1,4‐endoperoxide on melem unit typically functions as the active sites for H_2_O_2_ production.^[^ ^43^ ^]^ It is speculated that the introduction of larger amounts of Cr into CN framework could decrease the number of melem units, resulting in lower photocatalytic activity.

Several characteristic techniques were used to explore the enhancement origin of the photocatalytic performance of Cr‐SA‐CN. The time‐resolved photoluminescence (PL) spectrum is a direct proof of photogenerated carrier recombination.^[^ ^44^ ^]^ The average emission lifetime of Cr‐SA‐CN was 15.9 ns (Figure 2d), significantly higher that of pristine CN (5.3 ns), confirming that the introduction of Cr was beneficial for the utilization of photogenerated charge. Additionally, electrochemical impedance spectroscopy (EIS) was conducted under visible light soaking (Figure S7, Supporting Information). A marked decrease of Nyquist plots diameter for Cr‐SA‐CN was observed, indicative of lower charge transfer resistance.^[^ ^29^ ^]^ Rotating disk electrode (RDE) analysis was further performed to investigate the reaction pathway. The number of electron transfer involved in the O_2_ reduction was derived from the slope value of Koutecky—Levich plots. The estimated electron transfer number for Cr‐SA‐CN was closer to 2, revealing that the two‐electron O_2_ reduction was the predominant reaction for H_2_O_2_ generation (Figure 2e). The reusability of Cr‐SA‐CN was also studied using the recovered samples (Figure S8, Supporting Information). After five cycles of photocatalytic reaction, the formed amount of H_2_O_2_ still remained at 700 and 860 × 10^−6^ m in pure water and seawater, respectively, manifesting good photostability and recyclability of Cr‐SA‐CN. In summary, the incorporation of Cr single atoms improved the utilization efficiency of photogenerated carriers and thus significantly promoted efficient solar‐to‐H_2_O_2_ conversion.

### 2.3 Haloperoxidase‐Mimicking Activity

**Figure 3:**
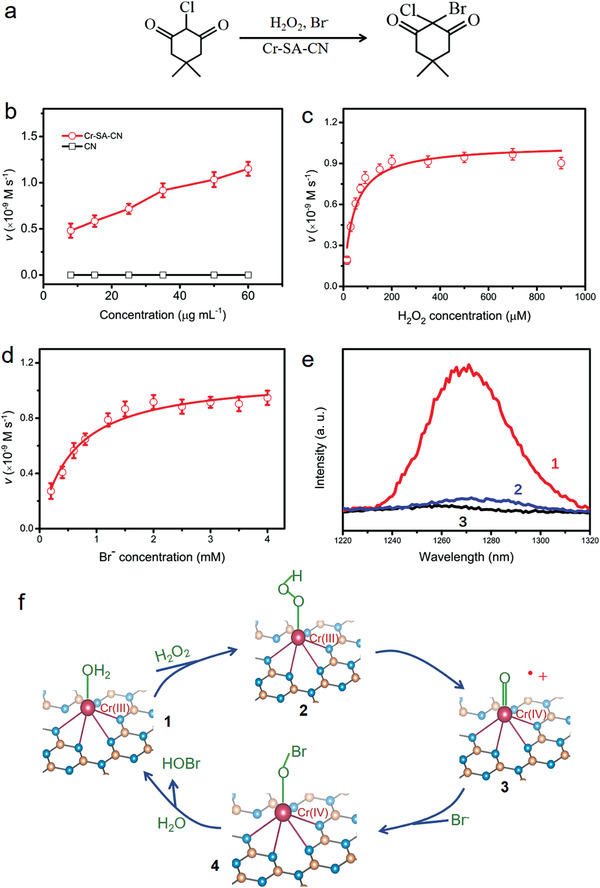
a) Reaction scheme of MCD bromination by Cr‐SA‐CN coupled with H_2_O_2_ and Br^−^ under dark. b) Concentration dependence of Cr‐SA‐CN and CN on catalytic bromination activity under dark in the presence of constant concentrations of Br^−^ (0.5 × 10^−3^ m ), H_2_O_2_ (35 × 10^−6^ m), and MCD (100 × 10^−6^ m) in 100 × 10^−3^ m Tris‐SO_4_ buffer (pH = 8.1). Kinetics of the bromination reaction of MCD as a function of c) H_2_O_2_ concentration (≈0–900 × 10^−6^ m) and d) Br^−^ concentration (≈0–4 × 10^−3^ m). e) Near‐infrared luminesce emission (1270 nm) of ^1^O_2_ (^1^Δ_g_) generation in 5 mL deuterium solvent in the presence of H_2_O_2_ and Cr‐SA‐CN: trace **1**, 0.05 m NH_4_Br; trace **2**, 0.05 m MCD; trace **3**, no NH_4_Br. f) Proposed catalytic cycle for Cr‐SA‐CN, 1: the initial state, 2: H_2_O ligand is exchanged by H_2_O_2_, 3: the intermediate state with an oxo‐chromium cation‐radical complex [(Cr(IV) = O)^•+^], 4: the intermediate state with a hypothetical chromium hypobromite adduct termed complex [Cr(IV)‐O‐Br].

Vanadium bromoperoxidases can efficiently catalyze the oxidation of a Br− into HOBr by H_2_O_2_.^[^ ^45^ ^]^ We used the bromination array of 2‐monochlorodimedone (MCD) in the presence of Br^−^ and H_2_O_2_ substrates in a Tris‐SO_4_ buffer solution (PH = 8.1) as a model system to qualitatively evaluate the intrinsic haloperoxidase‐mimicking activity of Cr‐SA‐CN under dark (**Figure** **3**a). The loss of extinction of the enolone band of MCD at 290 nm can be easily monitored spectrophotometrically, which was directly proportional to the catalytic activity of Cr‐SA‐CN.^[^ ^46^ ^]^ The bromination rate of MCD exhibited a linear dependence with the concentration of Cr‐SA‐CN, while pristine CN did not show any detectable catalytic activity (Figure 3b), demonstrating that the coordinated Cr single atoms played a dominating role in catalyzing HOBr generation. Furthermore, visible‐light illumination exerted a negligible effect on bromination rate, suggesting that the MCD bromination was not a photocatalytic reaction. The steady‐state reaction kinetic array was further performed by varying H_2_O_2_ or Br^−^ substrate concentration, while keeping the concentration of the remaining substrate constant. The steady‐state kinetic curves well followed the typical Michaelis–Menten behavior in the tested concentration range of H_2_O_2_ (Figure 3b) and Br^−^ (Figure 3c). Michaelis–Menten constant (*K*_m_) and maximum initial velocity (*V*_max_) were determined from the slope and intercept of the Lineaweaver–Burk double‐reciprocal plots. The calculated *K*_m_ values for H_2_O_2_ and Br^−^ were 44.7 × 10^−6^ m and 0.57 × 10^−3^ m, respectively. The lower *K*_m_ indicated the lower concentration of H_2_O_2_ required to reach the maximal activity, possibly due to the stronger affinity of melem units toward H_2_O_2_. Also, the *K*_m_ value of Cr‐SA‐CN for H_2_O_2_ and Br^−^ substrate failed within the same order of magnitude as those found for the natural vanadium bromoperoxidase (*K*_m_(H_2_O_2_) = 22 × 10^−6^ m, *K*_m_(Br^−^) = 18.1 × 10^−3^ m).^[^ ^47^ ^]^

An important diagnostic reaction for haloperoxidase‐mimicking activity is the HOBr assisted disproportionation of H_2_O_2_ to generate singlet molecular oxygen (^1^O_2_) in the absence of an organic scavenger.^[^ ^46^ ^]^ The formation of ^1^O_2_ can be monitored by chemiluminescence at 1270 nm owing to the transition of singlet oxygen (^1^O_2_, ^1^Δ_g_) to stable triplet (^3^O_2_, ^3^Σ_g_^−^). In the absence of Br^−^, no emission was detected in a deuterated solvent. A strong near‐infrared emission was observed in the presence of Cr‐SA‐CN, H_2_O_2_, and Br^−^ (Figure 3e), indicative of the formation of ^1^O_2_. After injecting excess MCD (0.1 m) into the solution, the chemiluminescence intensity was dramatically suppressed, further suggesting the intrinsic haloperoxidase‐like activity of Cr‐SA‐CN. To examine Cr element leaching, Cr‐SA‐CN was soaked in H_2_O_2_ and Br^−^ solution and stirred for over 1000 h, no detectable Cr was found in the resulting solution, revealing strongly anchored Cr single atoms.

To explore the catalytic mechanism of Br^−^ oxidation, we added the natural antioxidant cysteine to the solution containing Cr‐SA‐CN, Br^−^, H_2_O_2_, and MCD. It was found that the reaction rate of MCD bromination was completely hindered when 0.2 × 10^−3^ m cysteine scavenger was introduced, indicating that the generation of HOBr followed a radical mechanism.^[^ ^23^ ^]^ In addition, Cr(NO_3_)_3_ (0.5 × 10^−3^ m) was also incubated with the MCD solution under the same experimental conditions. No MCD bromination reaction was observed in the presence of the free Cr^3+^, indicating that the unique coordination structure of the Cr single atoms was responsible for the selective oxidation of Br^−^ substrate. With these results, a possible catalytic mechanism with Cr–N_4_ moiety functioned as a redox catalyst for the direct H_2_O_2_‐dependent oxidation of Br^−^ is proposed (Figure 3f), which is similar with that suggested for natural chloroperoxidase containing ferri‐protoporphyrin IX.^[^ ^21^, ^48^ ^]^ First, the H_2_O ligand adsorbed on the active site Cr–N_4_ (Cr(III)) nanozyme **1** is exchanged by H_2_O_2_ molecular, as revealed by low *K*_m_(H_2_O_2_). Then, the heterolytic cleavage of H_2_O_2_ resulted in the formation of intermediate state **3** with an oxo‐chromium cation‐radical complex [(Cr(IV) = O)^•+^], via the short‐lived intermediate state **2**. In a subsequent step, intermediate state **3** is unstable and supposed to interact with Br^−^ substrate to form a hypothetical chromium hypobromite adduct termed complex [Cr(IV)‐O‐Br]. Consequently, complex **4** decomposes to yield the initial state of nanozyme and allows the generation of HOBr.

**Figure 4:**
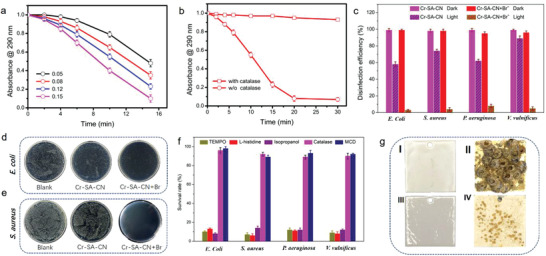
MCD bromination catalyzed by Cr‐SA‐CN with Br^−^ substrate in O_2_‐saturated Tris‐SO_4_ aqueous solution under visible light illumination (*λ* > 420 nm): a) with different Cr‐SA‐CN concentrations; b) with or without catalase scavenger. Reaction conditions: 0.1 mg mL^−1^ Cr‐SA‐CN, [Br^−^] = 1 × 10^−3^ m, [MCD] = 100 × 10^−6^ m, pH = 8.1. c) The disinfection efficiency of Cr‐SA‐CN or Cr‐SA‐CN+Br^−^ group under dark or visible light illumination. Representative digital images of d) *E. coli* and e) *S. aureus* coincubated with Cr‐SA‐CN and Cr‐SA‐CN+ Br^−^ for 1 h at 37 °C under visible light illumination. f) The disinfection performance of Cr‐SA‐CN+Br^−^ under visible light illumination with different scavengers. g) Digital images of stainless‐steel plates painted with different additives: fresh plates with (I) CN and (III) Cr‐SA‐CN; fouling plates with (II) CN and (IV) Cr‐SA‐CN after immersing in seawater for 52 days.

We then explored whether the in situ generated H_2_O_2_ can be furthered to combine with Br^−^ to generate HOBr. We first examined the absorbance variations of MCD because light illumination could result in the partial degradation of MCD (Figure S9, Supporting Information), and then took this as a benchmark to eliminate the effect of light illumination on MCD. Control experiments in N_2_‐saturated aqueous solution or in absence of Br^−^ under light soaking did not exhibit detectable changes in absorption (Figure S10, Supporting Information). **Figure** **4**a shows the absorption of MCD over time in the presence of Cr‐SA‐CN and Br^−^ in O_2_‐saturated aqueous solution under visible‐light illumination (*λ* > 420 nm). Notably, the absorption at 290 nm gradually decreased with increased Cr‐SA‐CN concentrations, implying the occurrence of cascade reactions for MCD bromination. It should be noted that the absorption decreased slowly in the first 4 min and then rapidly decayed within next 10 min. Since the concentration of H_2_O_2_ intermediate produced from water and O_2_ was relatively low at the initial stage, restricting the catalytic oxidation rate of Br^−^ substrate. The prolonged light irradiation provided continuous and sufficient H_2_O_2_ intermediate, which would accelerate the HOBr formation from H_2_O_2_ and Br^−^ and thus result in rapid MCD bromination. Such cascade reaction under light illumination was also similar with MCD bromination catalyzed by Cr‐SA‐CN under dark with additional H_2_O_2_ (Figure S11, Supporting Information). To reveal the origin of the MCD bromination under visible light irradiation, we examined the absorption decay with H_2_O_2_ scavenger (Figure 4b). The presence of catalase scavenger yielded negligible absorption variation of MCD, indicating that the direct photosynthesis of HOBr was very limited over Cr‐SA‐CN.^[^ ^49^ ^]^ These results confirmed that the cascade reactions were successfully realized on Cr‐SA‐CN under visible light irradiation, that is, the in situ photocatalytic H_2_O_2_ generation and subsequently the utilization of H_2_O_2_ intermediate for HOBr formation by haloperoxidase‐mimicking reaction.

### 2.4 Cr‐SA‐CN for Biofilm Prevention

Next, the biofilm inhibition capability of Cr‐SA‐CN was evaluated by investigating the inactivation efficiencies of several typical bacteria, i.e., *Staphylococcus aureus* (*S. aureus*), *Escherichia coli* (*E. Coli*), *Pseudomonas aeruginosa* (*P. aeruginosa*), and marine bacteria *Vibrio vulnificus* (*V. vulnificus*). Visible light alone exerted a minor antibacterial activity (Figure S12, Supporting Information). Similarly, the disinfection activity of Cr‐SA‐CN or Cr‐SA‐CN+Br^−^ group was negligible in dark (Figure 4c). By contrast, Cr‐SA‐CN with or without Br^−^ substrate obtained a moderate antibacterial ability under visible light illumination, and Cr‐SA‐CN+Br^−^ yielded significantly higher biocidal activity than Cr‐SA‐CN. The disinfection efficiency of Cr‐SA‐CN+Br^−^ was 97%, 96%, 92%, and 95% for *E. coli*, *S. aureus*, *P. aeruginosa*, and *V. vulnificus*, respectively, which was also comparable with that of Cr‐SA‐CN+Br^−^+H_2_O_2_ reference group in dark (Figure S13, Supporting Information). Figure 4d,e shows the digital images of *E. coli* and *S. aureus* after incubation on cultural plates, respectively. Blank control without any additives showed a dense bacterial population. Cr‐SA‐CN+Br^−^ groups exhibited an obvious decrease in the number of bacterial colonies, further verifying that the cascade system of Cr‐SA‐CN synergized its catalyzation to realize superior bacteria‐targeting ability.

Reactive oxygen species (ROS) could be nonspecifically produced on semiconducting catalysts from water and O_2_ under light irradiation.^[^ ^50^ ^]^ To prove the disinfection mechanism of Cr‐SA‐CN directly, the disinfection contribution of each ROS was examined through a series of scavenger quenching experiments. Scavengers 2,2,6,6‐tetramethylpiperidinooxy (TEMPO), *L*‐histidine, isopropanol, catalase, and MCD, were chosen to quench superoxide (•O^2−^), ^1^O_2_, hydroxyl radical (OH•), H_2_O_2_, and HOBr, respectively.^[^ ^51^ ^]^ The presence of TEMPO, *L*‐histidine, and isopropanol scavengers in Cr‐SA‐CN+Br^−^ bacterial suspension did not show obvious impact on bacterial inactivation (Figure 4f). In sharp contrast, both catalase and MCD exerted strong suppression on inactivating bacteria, indicating that the disinfection efficiency strongly depended on H_2_O_2_ and HOBr generation. However, H_2_O_2_ yielded relatively poor antibacterial ability (Figure 4c). Thus, the strong quenching effect of catalase scavenger could be ascribed to the depletion of photogenerated H_2_O_2_ intermediate, which subsequently disturbed the continuous HOBr formation from H_2_O_2_ and Br^−^. All these evidences strongly indicate that biocidal HOBr dominated the disinfection activity and showed the highest contribution on antibacterial ability.

Since Cr‐SA‐CN also exhibited the superior photoactivity for H_2_O_2_ generation in seawater (PH = ≈8.1–8.3), providing favorable conditions for marine biofilm prevention on Cr‐SA‐CN by utilizing natural occurring Br^−^ substrate in seawater (≈0.8 × 10^−3^ m). The antibiofilm performance of Cr‐SA‐CN was assessed by exposing the stained‐steel plates (2×2 cm^2^) painted with soft paint formulations containing 5 wt% Cr‐SA‐CN moiety to natural seawater in open ocean. After immersing in natural seawater for 52 days, CN‐containing plates were densely covered with barnacle and algae (Figure 4g), which was similar with the control plates without any antibiofouling moiety (Figure S14, Supporting Information). As expectedly, samples containing Cr‐SA‐CN showed remarkable antibiofouling efficiency, with obviously decreased marine microorganism attachment. All these findings indicate that the cascade processes (H_2_O_2_ production and HOBr formation) simultaneously occurring on Cr‐SA‐CN nanoplatform possesses potential for combating biofilm.

## 3 Conclusion

In summary, we have rationally constructed a Cr single atom nanozyme with the specific capability to catalyze cascade reaction for combating biofouling. Benefitting from atomically dispersed Cr single atoms, Cr‐SA‐CN induced H_2_O_2_ photosynthesis and haloperoxidase‐mimicking activity. The bifunctional Cr‐SA‐CN nanoplatform promoted sustainable HOBr formation under visible light radiation, resulting in superior antibacterial ability. Real field tests in seawater indicated that Cr‐SA‐CN as an antimicrobial additive of coating enabled an inert surface against the colonization of marine microorganisms. Crucial for the success of Cr‐SA‐CN in thwarting biofilm resisted in the in situ self‐supply of photogenerated H_2_O_2_ for the subsequent haloperoxidase‐mimicking reaction in a sequential manner. This study not only demonstrates the ability of single atom nanozymes in combating biofouling but also provides a strategy for constructing more innovative nanozymes with multifunctionality for a variety of synergistic biomimicking processes.

## Conflict of Interest

The authors declare no conflict of interest.

## Supporting information

Supporting Information

## Data Availability

The data that support the findings of this study are available from the corresponding author upon reasonable request.
