# Supplementary material for: Atomic Chromium Coordinated Graphitic Carbon Nitride for Bioinspired Antibiofouling in Seawater
Source: Adv Sci (Weinh). 2022 Jan 20;9(8):2105346. doi: 10.1002/advs.202105346 (PMC8922116; doi:10.1002/advs.202105346)
Supplement: Supplementary file 1 — Supporting Information [file ADVS-9-2105346-s001.pdf]

ADVANCED SCIENCE
Open Access

Supporting Information

for *Adv. Sci.*, DOI 10.1002/advs.202105346

Atomic Chromium Coordinated Graphitic Carbon Nitride for Bioinspired Antibiofouling in Seawater

*Qiang Luo, Yilan Li, Xiaobing Huo, Linqian Li, Yinqiao Song, Shipeng Chen, Hong Lin and Ning Wang**

WILEY-VCH

ADVANCED SCIENCE
Open Access

Supporting Information

for *Adv. Sci.*, DOI: 10.1002/advs.202105346

Atomic Chromium Coordinated Graphitic Carbon Nitride for Bioinspired Anti-biofouling in Seawater

*Qiang Luo, Yilan Li, Xiaobin Huo, Linqian Li, Yinqiao Song, Shipeng Chen, Hong Lin, Ning Wang**

Supporting Information

Atomic Chromium Coordinated Graphitic Carbon Nitride for Bioinspired Anti-biofouling in Seawater

*Qiang Luo, Yilan Li, Xiaobin Huo, Linqian Li, Yinqiao Song, Shipeng Chen, Hong Lin, Ning Wang**

Experimental Section

Preparation of Cr-SA-CN: For the synthesis of Cr-SA-CN, 5 mmol melamine was dissolved in 80 mL dimethyl sulfoxide followed by stirring for approximately 30 min at room temperature, then chromium (III) nitrate nonahydrate dissolved in 5 mL dimethyl sulfoxide was slowly added into melamine solution. The resulting mixture was then heated at 60 °C under continuous magnetic stirring. Subsequently, 5 mmol cyanuric acid dissolved in 50 mL dimethyl sulfoxide was dropwise slowly added to the heat solution using a syringe with the total volume approaching about 60 mL. The white precipitate was formed immediately upon the addition of cyanuric acid solution. The resulting precipitate was collected and washed with ethanol several times. The solid precursor was then calcined at 550 °C for 8 h under N_2 atmosphere. For the preparation of pristine carbon nitride, the similar procedure was used but without the addition of Cr precursor.

Photocatalytic production of H_2O_2: 50 mg of nanozyme was ultrasonically dispersed in 50 mL deionized water or real seawater in a 100 mL borosilicate glass bottle without the addition of sacrificial agent. Under mild magnetic stirring, O_2 was bubbled into the aqueous solution for 30 min to achieve equilibrium. The photocatalytic reaction was performed by exposing the sealed reaction system to visible light using a solar simulator (AM 1.5 G, 100 mW cm^{-2}, Oriel, USA) with a 420 nm cutoff filter. The reaction was maintained at 25±0.5 ^{o}C. During the photoirradiation, 2 mL solution was taken at different time intervals and filtered to remove the photocatalyst. The yield of H_2O_2 was measured by HPLC in combination with an electrochemical analyzer or using a standard iodimetry analysis method.[29,40] For stability test, the nanozyme was recovered after each reaction by centrifugalizing and washed with deionized water, then the sample was reused for photocatalytic reaction (8 h for each cycle).

For photoelectrochemical measurements, electrochemical impedance spectroscopy (EIS) was conducted in 0.1 M KCl solution under light illumination (λ≥420 nm) in a three-electrode configuration on a Zahner electrochemical system (Zahner-Electrik GmbH&Co. KG,

Germany). Linear sweep voltammetry on a rotating disk electrode (RDE) was also performed with the similar method. The Nyquist plots were obtained at a bias of 0.7 V in the frequency range of 1Hz to 100 kHz. The catalysts deposited on FTO substrates by spin coating were applied as the working electrode. Ag/AgCl and Pt wire were used as reference electrode and counter electrode, respectively. The average electron transfer number for O_2 reduction reaction was determined according to the slopes of Koutecky-Levich plots.

The Mott-Schottky plots were also investigated by using a conventional three-electrode cell. The working electrodes were prepared by depositing Cr-SA-CN photocatalysts on fluorine doped tin oxide coated glass. Ag/AgCl and Pt wire were used as the reference electrode and reference electrode, respectively. The measurements were carried out in 0.2 M Na_2SO4 aqueous solution electrolyte (pH=7.1, 25 ^oC).

Haloperoxidase-like activity mimic and reaction kinetics: The catalytic activity of the Cr-SA-CN in the bromination of MCD were performed for 4 min at 25±0.5 ^oC in 100 mM Tris-SO_4 buffer (pH 8.1) in dark. Data analysis was performed by evaluating the UV-vis absorpation changes at 290 nm. The initial oxidation rate of Br^- substrate was extracted from the slope of the absorbance-time curve (-dA_{290}/dt). For the steady-state reaction kinetic evaluation, the concentration of H_2O_2 was varied at a range of 0~500 μM in the presence of constant concentrations of Cr-SA-CN (0.05 mg mL^{-1}), Br^- (0.5 mM), and MCD (100 μM). The concentrations of Br^- were varied from 0~5 mM with constant concentrations of Cr-SA-CN (0.035 mg mL^{-1}), H_2O_2 (30 μM), and MCD (100 μM). The Michaelis-Menten constant (K_m) was obtained by Lineweaver-Burk linearization.

The $^1O_2$ production was monitored by chemiluminescence from the spin-forbidden transition emission at 1270 cm^{-1} in Tris-SO_4 buffer (pH 8.1). Reactions were triggered and performed by adding 10 mM H_2O_2 into a 5 mL deuterium oxide suspension containing 20 mg Cr-SA-CN and 0.05 M NH_4Br. Reactions also were performed with MCD or in the absence of the NH_4Br substrate. The near-infrared emission spectra were continuously collected from

1220 and 1320 nm.

Enzymatic cascade reaction: 2 mg photocatalyst was added into 20 mL Tris-SO_4 buffer solution (pH 8.1) containing 1 mM NH_4Br substrate and 100 µM MCD indicator. After 30 min O_2-purging in dark, the reaction system was exposed to visible light (λ≥420 nm) at 25±0.5 ^oC. The resulting reaction solution was traced by UV-Vis spectroscopy after filteration.

Antibacterial assay: Bacteria *E. coli, S. aureus, P. aeruginosa* and *V. vulnificus* were used as the model bacteria to evaluate the antibacterial ability of Cr-SA-CN. The exponential growth bacteria were transferred and incubated in fresh Luria-Bertani (LB) medium with different additives (Cr-SA-CN (0.1 mg mL^{-1}) and/or Br^- (1 mM)) at 37 ^oC with moderate shaking. The solar-disinfection performance was investigated by photoirridiating the bacterial solution using a solar simulator (AM 1.5G illumination) equipped with a 420 nm filter. After 60 min photoillumination, the bacterial concentrations was evaluated using the standard spread-plating techniques. Subsequently, 100 µl of bacteria was plated on agar-LB medium and left to grow for overnight at 37 ^oC for 12 h. The disinfection performance was determined by the concentration ratio of bacteria. All experiments were performed three times.

In situ marine field tests: The real field test was performed on July-September at Haikou bay in Haikou, China. The stainless-steel plates (2 cm × 2 cm) were ultrasonically washed with detergent, deionized water, and acetone successively. The Cr-SA-CN with a dry weight of about 3.5 wt.% was added into the abrasive rosin-based formulation paint. After homogenization, the paint was applied onto the clean stainless-steel plates. The painted plates without additional additives were also used for comparison. The painted substrates were fixed to a stationary experimental raft and were immersed into seawater at a depth of ~1 m.

Characterization: XRD patterns were collected on a D8 ADVANCE diffractometer at a voltage of 40 kV using a Cu Kα radiation source (Bruker, Germany). N_2 adsorption-desorption measurements were conducted on an ASAP 2460 physical adsorption

instrument (Micromeritics, USA), and the specific surface area was determined using the Brunauer-Emmett-Teller (BET) method. SEM images were measured with a LEO 1530 scanning electron microscopy at an accelerating voltage of 20 kV (Zeiss, Germany). TEM observations were carried out by a JEM-2010 microscope (JEOL, Japan). The atomic-resolution HAADF-STEM images were obtained on an ARM-200CF STEM equipped with double spherical aberration correctors and energy-dispersive X-ray spectroscopy (EDS). UV-vis diffuse reflectance spectra were recorded on a Shimadzu UV-2550 spectrophotometer. Fourier transform infrared (FTIR) spectra of the as-prepared catalysts were obtained on an FTIR-650 spectrometer. XPS were characterized using a ESCALAB 250Xi spectrometer (Thermo Fisher Scientific Inc., USA) with an aluminum anode X-ray source. The XAFS spectra were obtained on the beamline BL01C1 in NSRRC (Shanghai, China) with a Silicon (111) double-crystal monochromator. The acquired XANES and EXAFS data was processed by Athena module implemented in the IFEFFIT software packages. The optical absorption was collected on a UV-vis spectrometer (UV-1900i, Shimadzu). The time-resolved photoluminescence emission spectra were obtained on a FLS920 fluorescence spectrometer with an excitation wavelength of 375 nm (Edinburgh Instruments, UK).

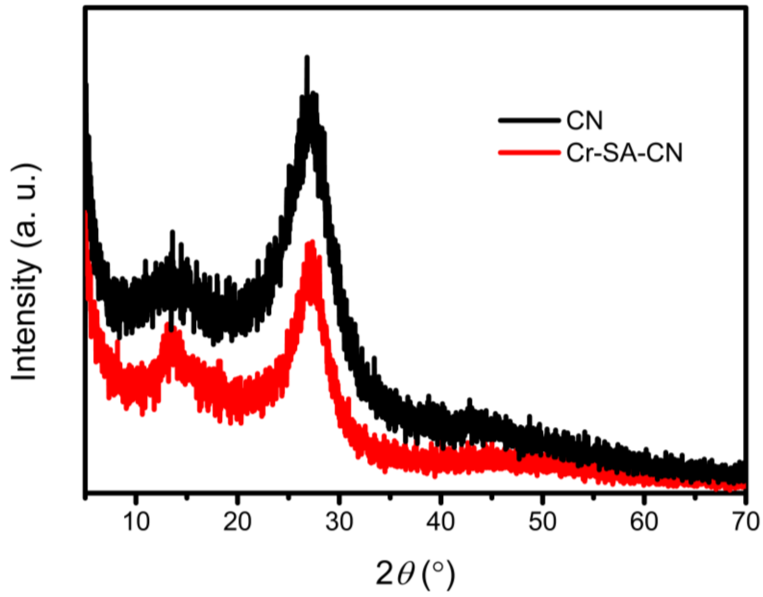

Figure S1. XRD patterns of the as-synthesized Cr-SA-CN and reference CN.

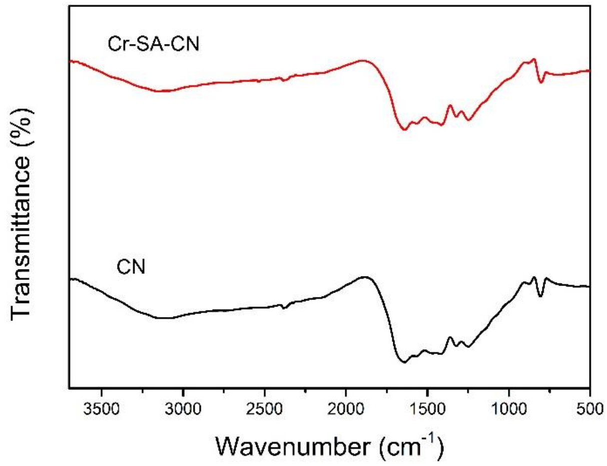

Figure S2. Typical IR spectra of CN and Cr-SA-CN samples.

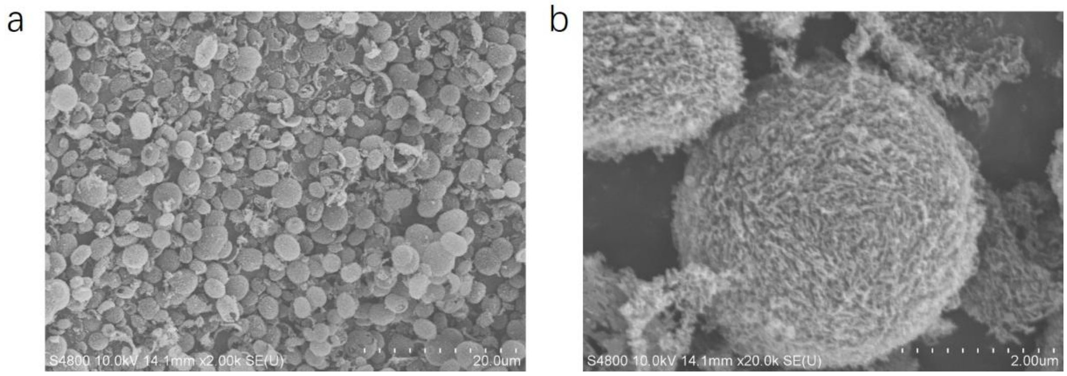

Figure S3. Typical surface SEM images of Cr-SA-CN sample.

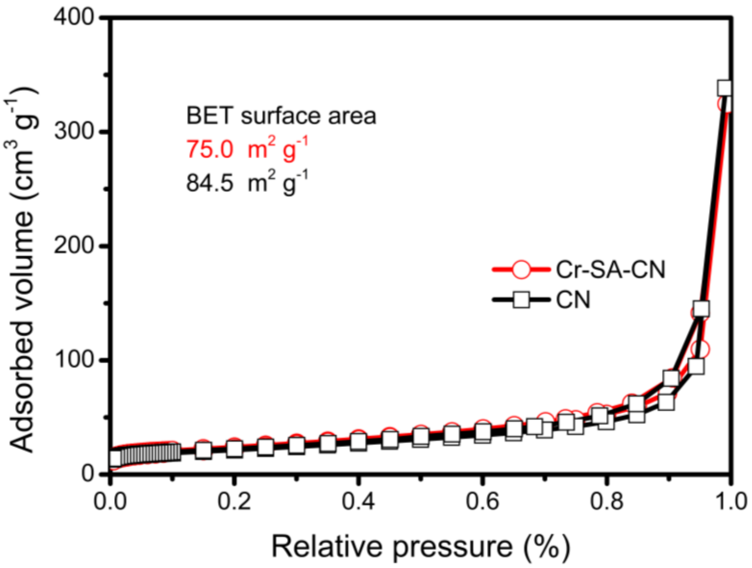

Figure S4. N_2 adsorption-desorption curves of CN and Cr-SA-CN.

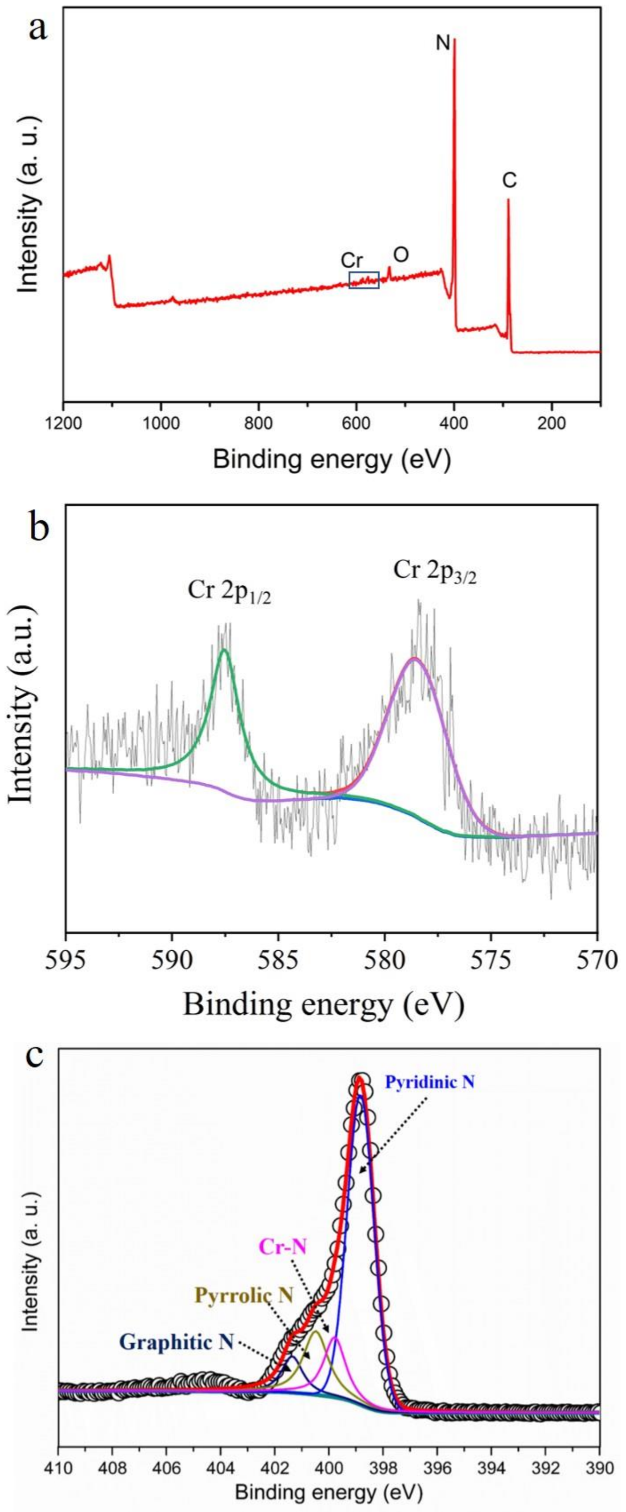

Figure S5. (a) Full XPS spectrum of Cr-SA-CN, High-resolution Cr 2p (b) and N 1s XPS spectrum of Cr-SA-CN.

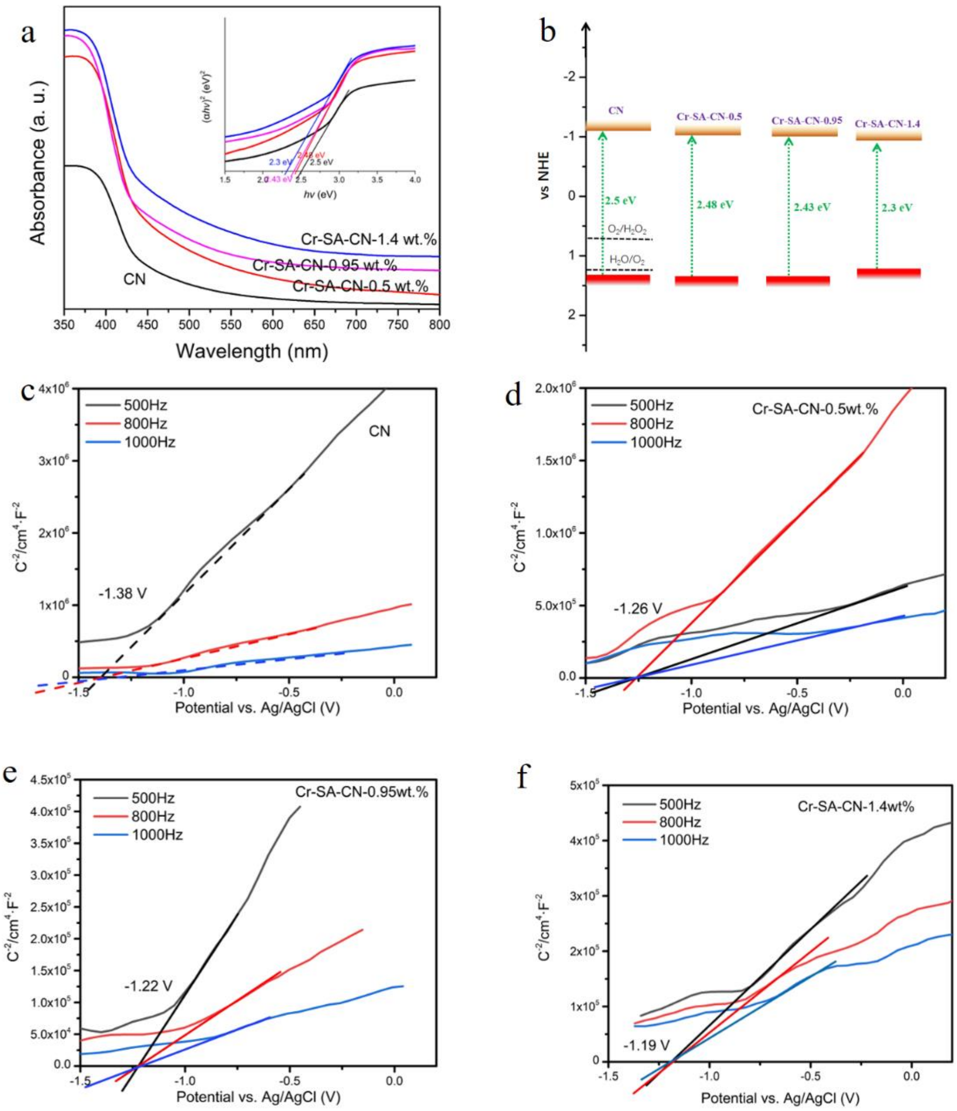

Figure S6. (a) UV-Vis diffuse reflectance spectra and corresponding Taus plots (inset). (b) Schematic band diagram of of Cr-SA-CN with different Cr amount (0.5, 0.9, 1.4 wt.%). (c-f) Mott-Schottky plots of g-C_3N_4 with different Cr amount (0.5, 0.9, 1.4 wt.%).

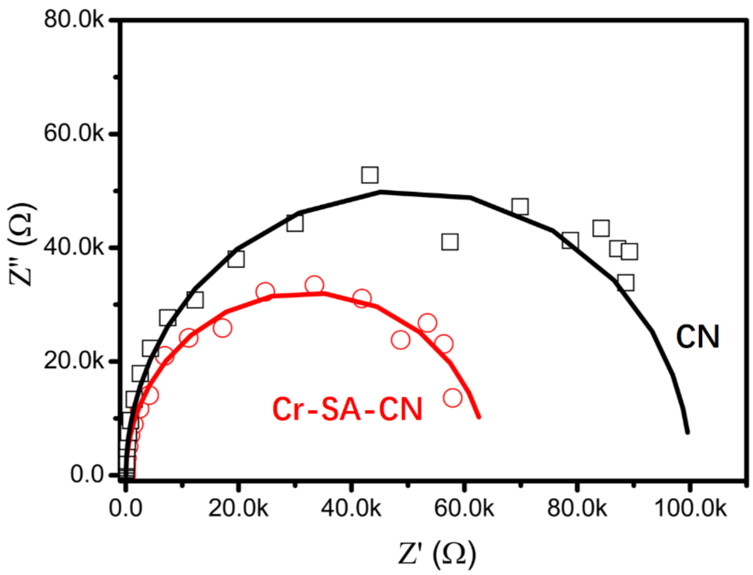

Figure S7. Electrochemical impedance spectroscopy (EIS) Nyquist plots of CN and Cr-SA-CN measured in 0.1 M KCl under visible light at a bias of 0.7 V (versus Ag/AgCl electrode) in the frequency range of 1Hz to 100 kHz.

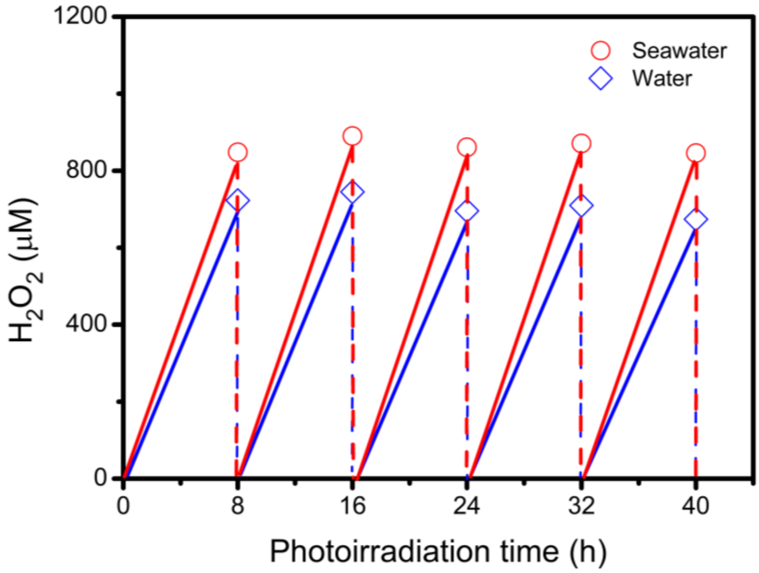

Figure S8. Reusability of CN and Cr-SA-CN on H_2O_2 photogeneration after 8 h under AM1.5G simulated sunlight irradiation (λ>420 nm).

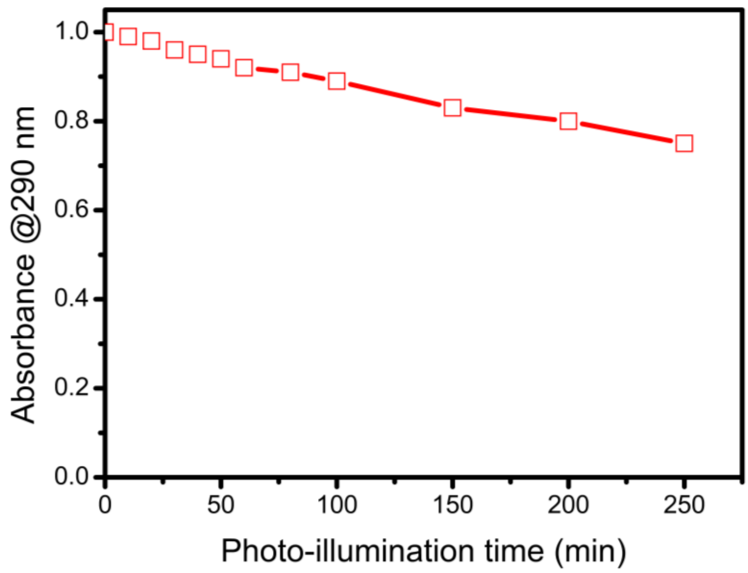

Figure S9. The degradation of MCD under visible-light irradiation (AM 1.5G, 100 mW cm^{-2}, λ>420 nm).

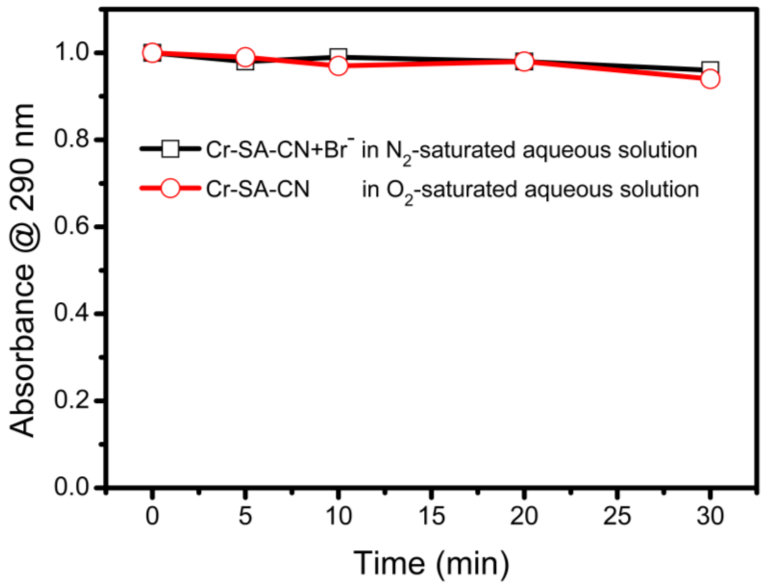

Figure S10. MCD bromination in N_2-saturated aqueous solution or in absence of Br^- in O_2-saturated aqueous solution under light irradiation (AM 1.5G, 100 mW cm^{-2}, λ>420 nm).

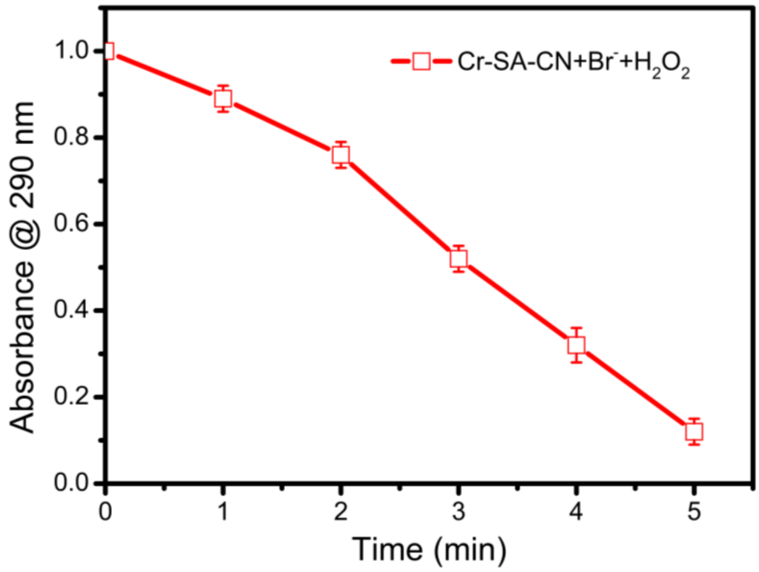

Figure S11. MCD bromination under dark in the presence Cr-SA-CN, Br^- and H_2O_2.

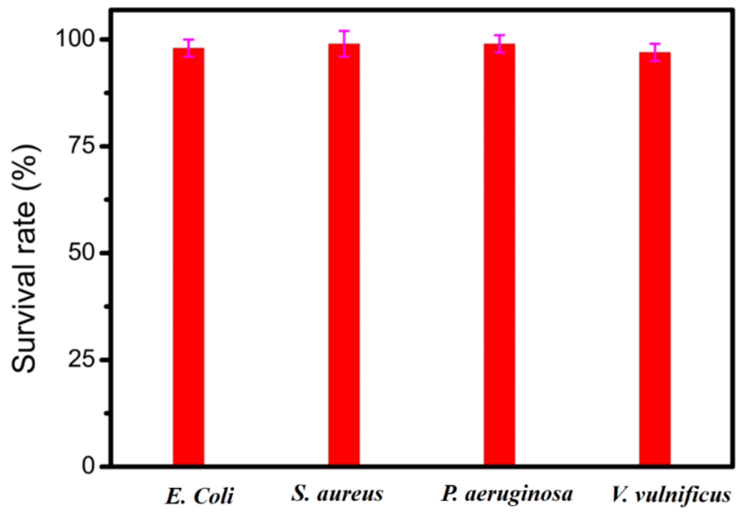

Figure S12. The survival rate of different bacteria under visible-light irradiation in presence of any additive (AM 1.5G, 100 mW cm^{-2}, λ>420 nm).

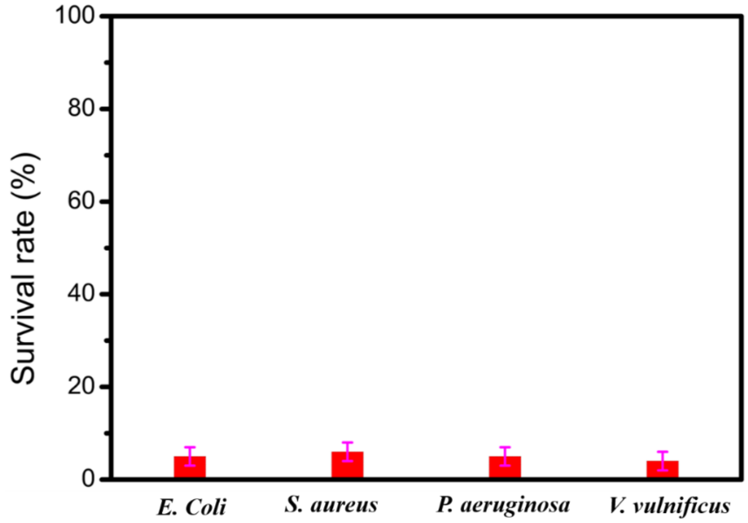

Figure S13. The antibacterial ability of Cr-SA-CN toward different bacteria in the presence of Br^- and additional H_2O_2 under dark.

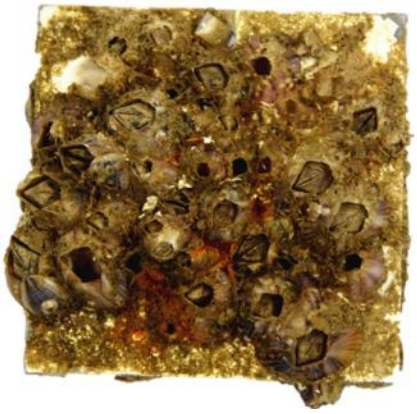

Figure S14. Digital images of reference painted stainless-steel plates without additives.

Table S1. EXAFS fitting parameters at the Cr K-edge for various samples (S_0^2=0.837)

Sample	Shell	N^a	R(Å)^b	σ^2(Å^2)^c	ΔE_0(eV)^d	R factor
Cr-foil	Cr-Cr	8	2.49	0.0061	3.4	0.0026
	Cr-Cr	6	2.87	0.0047		
Cr_2O_3	Cr-O	6.0	1.99	0.0047	-3.7	0.0008
	Cr-Cr	4.0	2.94	0.0138		
Cr-SA-CN	Cr-N	4.5	2.01	0.0018	-1.1	0.0033

$^a N$: coordination numbers; $^b R$: bond distance; $^c \sigma^2$: Debye-Waller factors; ^{d} ΔE_0: the inner potential correction. R factor: goodness of fit. S_0^2 was set to 0.837, according to the experimental EXAFS fit of Cr foil by fixing CN as the known crystallographic value.
